# Dissecting EXP2 sequence requirements for protein export in malaria parasites

**DOI:** 10.3389/fcimb.2023.1332146

**Published:** 2024-01-12

**Authors:** Ethan L. Pitman, Natalie A. Counihan, Joyanta K. Modak, Mrittika Chowdury, Paul R. Gilson, Chaille T. Webb, Tania F. de Koning-Ward

**Affiliations:** ^1^ School of Medicine, Deakin University, Geelong, VIC, Australia; ^2^ Institute for Mental and Physical Health and Clinical Translation (IMPACT), Deakin University, Geelong, VIC, Australia; ^3^ Burnet Institute, Melbourne, VIC, Australia; ^4^ Department of Microbiology and Immunology, University of Melbourne, Parkville, VIC, Australia; ^5^ Biomedicine Discovery Institute and Department of Microbiology, Monash University, Clayton, VIC, Australia; ^6^ Centre to Impact AMR, Monash University, Clayton, VIC, Australia

**Keywords:** *Plasmodium*, PTEX, protein export, GRA17, nutrient exchange, EXP2

## Abstract

Apicomplexan parasites that reside within a parasitophorous vacuole harbor a conserved pore-forming protein that enables small-molecule transfer across the parasitophorous vacuole membrane (PVM). In *Plasmodium* parasites that cause malaria, this nutrient pore is formed by EXP2 which can complement the function of GRA17, an orthologous protein in *Toxoplasma gondii.* EXP2, however, has an additional function in *Plasmodium* parasites, serving also as the pore-forming component of the protein export machinery PTEX. To examine how EXP2 can play this additional role, transgenes that encoded truncations of EXP2, GRA17, hybrid GRA17-EXP2, or EXP2 under the transcriptional control of different promoters were expressed in EXP2 knockdown parasites to determine which could complement EXP2 function. This revealed that EXP2 is a unique pore-forming protein, and its protein export role in *P. falciparum* cannot be complemented by *T. gondii* GRA17. This was despite the addition of the EXP2 assembly strand and part of the linker helix to GRA17, which are regions necessary for the interaction of EXP2 with the other core PTEX components. This indicates that the body region of EXP2 plays a critical role in PTEX assembly and/or that the absence of other *T. gondii* GRA proteins in *P. falciparum* leads to its reduced efficiency of insertion into the PVM and complementation potential. Altering the timing and abundance of EXP2 expression did not affect protein export but affected parasite viability, indicating that the unique transcriptional profile of EXP2 when compared to other PTEX components enables it to serve an additional role in nutrient exchange.

## Introduction

The ability of *Plasmodium* parasites to remodel their host red blood cell (RBC) is central to their ability to evade the host immune response and cause the pathological symptoms of the disease malaria. In 2021 alone, ~619, 000 people died as a result of a *Plasmodium* infection, with *P. falciparum* being the most lethal *Plasmodium* spp. (WHO, 2021). The ability of *P. falciparum* to remodel its host RBC is underpinned by the export of many hundreds of parasite proteins into the host cell that lead to perturbation of the physiochemical properties of the cell (de Koning-Ward et al., 2016). This includes changes to the deformability and permeability properties of the host cell as well as presentation of the major virulence factor *P. falciparum* Erythrocyte Membrane Protein 1 (PfEMP1) on the RBC surface to facilitate cytoadhesion of the infected RBC to the microvascular endothelium to prevent splenic clearance (Maier et al., 2008; Spillman et al., 2015; de Koning-Ward et al., 2016; Wahlgren et al., 2017).

To access the host cell, *Plasmodium* proteins secreted into the encasing parasitophorous vacuole (PV) traverse the PV membrane (PVM) via the *Plasmodium* translocon of exported proteins (PTEX) (de Koning-Ward et al., 2009). This protein-conducting channel comprises three core components, namely, EXP2, PTEX150, and Heat shock protein 101 (HSP101) (de Koning-Ward et al., 2009; Ho et al., 2018), which are essential for protein export and parasite survival (Matthews et al., 2013; Matz et al., 2013; Beck et al., 2014; Elsworth et al., 2014; Kalanon et al., 2015; Charnaud et al., 2018; Garten et al., 2018). In addition, PTEX comprises two auxiliary components, namely, PTEX88 and TRX2, that contribute to parasite virulence although they are not critical for PTEX function and survival (Matz et al., 2015; Chisholm et al., 2016). The channel-forming constituent of PTEX that spans the PVM is formed by seven protomers of EXP2 that were shown by cryo-electron microscopy to form a funnel-shaped oligomer (Ho et al., 2018). It is the N-terminus of each of EXP2 protomer that forms an amphipathic transmembrane helix that spans the PVM, whereas the remaining C-terminal bulk of the protomers project out into the PV space. The so-called “assembly strand” of EXP2 located at its C-terminal end (D231-S234) projects upward in some protomers to form contacts with HSP101. In between the adjacent EXP2 protomers are seven protomers of PTEX150 that oligomerize to form a pseudo-symmetrical flange-shaped heptamer at the mouth of the EXP2 funnel (Ho et al., 2018). PTEX150 provides a scaffold for EXP2 and the other core PTEX component, HSP101, to interact (Elsworth et al., 2016; Ho et al., 2018). HSP101 is a ClpB AAA^+^-ATPase and member of the Clp/HSP100 family of chaperones that forms a ring-shaped hexamer (El Bakkouri et al., 2010). It serves as the PTEX unfoldase, utilizing ATP to unfold cargo so they are competent for export, and it drives protein translocation across the PVM through the EXP2 pore (Matthews et al., 2019).

EXP2 has also been shown to have a function independent of PTEX (Garten et al., 2018). Garten et al. were able to demonstrate that a large proportion of *P. falciparum* EXP2 does not co-purify with the rest of PTEX and instead serves as a nutrient pore to facilitate the transfer of small molecules across the PVM. The dual function of EXP2 potentially explains its distinct expression profile in comparison to the other core constituents of PTEX; peak expression of EXP2 occurs in trophozoite stages, whereas peak expression of HSP101 and PTEX150 occurs in late schizont/early ring stages (Otto et al., 2010; Bullen et al., 2012). Interestingly, the nutrient pore function of EXP2 is conserved across Apicomplexan parasites that reside in a PV and indeed EXP2 can complement the function of the *Toxoplasma gondii* EXP2 ortholog, GRA17 (Gold et al., 2015). However, *T. gondii* does not utilize GRA17 for export of proteins into its host cell, and orthologs of PTEX150 and HSP101 are absent, and, instead, translocation of effector proteins in *T. gondii* is mediated by the MYR complex at the PVM (Franco et al., 2016; Marino et al., 2018).

In this study, we sought to address how EXP2 can facilitate both protein export and nutrient transfer in *P. falciparum*. We hypothesized that EXP2 can complement the function of GRA17 in *T. gondii* because EXP2 can form pores in the PVM to still permit nutrient exchange but that GRA17 would not be able to complement the loss of EXP2 in *P. falciparum* because GRA17 lacks the necessary sequences to assemble into the PTEX complex. To test this and examine whether the EXP2 assembly strand was the only critical sequence required for docking of PTEX150 and HSP101, we examined whether *P. falciparum* EXP2 knockdown parasites could be complemented with a variety of expression constructs, including GRA17 and GRA17 fused to sequences unique to EXP2. We also examined the impact on parasites after altering the temporal expression of EXP2 to mimic that of other PTEX components to identify why EXP2 has a unique expression profile. These studies reveal that sequences beyond the EXP2 assembly strand and linker helix are required for assembly into the PTEX complex to facilitate protein export and that EXP2 has a unique transcriptional profile because it also essential for nutrient exchange.

## Materials and methods

### Plasmid constructs

Several complementation constructs were created for this study, all of which contained 3× cMyc epitope tags and a blasticidin-S deaminase selectable marker to enable selection of transfectants. For expression of *P. falciparum* EXP2 under the *exp2* promoter, the *exp2* coding sequence (CDS) and ~1.5 kb of sequence immediately upstream was amplified from *P. falciparum* 3D7 genomic DNA using oligonucleotides DO753 and DO891. The resulting product was digested with *Sal*I and *Age*I and inserted into the corresponding sites of a modified version of pHGBrHrBl-12 (a gift from Danny Wilson), in which the *gfp* CDS had been replaced with 3× cMyc epitope tags (pHSP865′-cMyc) to create pEXP2 5′-EXP2-cMyc. For the expression of *exp2* under the *hsp86* promoter, the *exp2* CDS was amplified with oligonucleotides DO757 and DO891, digested with *Bgl*II and *Age*I and ligated immediately downstream of the *hsp86* promoter in the corresponding sites of pHSP865′-cMyc to create pHSP86 5′-EXP2-cMyc. To create pEXP2ΔAT-cMyc, *exp2* was amplified using oligonucleotides DO757 and DO1115, resulting in a 216-bp C-terminal truncation and cloned in place of wild-type *exp2* in pHSP86 5′-EXP2-cMyc. For GRA17 expression in *P. falciparum*, a *T. gondii gra17* codon optimized sequence was synthesized by GeneScript. The *gra17* sequence harbored a 171-bp N-terminal truncation when compared to that of TGME49_222170 in ToxoDB (Gajria et al., 2008) based on previous evidence that indicates the CDS of GRA17 commences at amino acid 58 (MRAIRSVV…) (Gold et al., 2015). This modified *gra17* sequence was then substituted for *exp2* in pHSP86 5′-EXP2-cMyc to create pGRA17-cMyc. To create pGRA17+AT-cMyc, the last 216 bp of *exp2* CDS (corresponding to amino acids 216–287) was amplified using oligonucleotides DO862 and DO863 and inserted in frame at the C-terminus of *gra17* in pHSP86 5′-EXP2-cMyc using restriction enzyme sites *Age*I and *Nhe*I. Expression of EXP2 was also placed under either the *resa* or *ptex150* promoter. In this case, oligonucleotides DO1191/DO1192 and DO1481/DO1482 were used to amplify the *resa* and *ptex150* promoters, respectively, and the resulting products substituted for the *hsp86* promoter in pHSP86 5′-EXP2-cMyc using restriction enzymes *Sal*I and *Bgl*II. The resulting plasmids were termed pRESA5′-EXP2-cMyc and pPTEX150 5′-EXP2-cMyc. All oligonucleotides used for polymerase chain reaction (PCR) amplification are provided in **Supplementary Table 1**.

### Culture and transfection of *P. falciparum*

*P. falciparum* (3D7) parasites were cultured in O^+^ human RBC obtained from the Australian Red Cross at 4% hematocrit in media comprising RPMI 1640 medium (Life Technologies), 25 mM 4-(2-hydroxyethyl)-1-piperazineethanesulfonic acid (HEPES) (Astral Scientific), gentamicin (20 mg/L; Life Technologies), hypoxanthine (50 mg/L; Sigma-Aldrich), 25 mM sodium bicarbonate (Sigma-Aldrich), and 0.5% (w/v) Albumax (Life Technologies). Cultures were maintained at 37°C under an atmosphere of 5% CO_2_ and 1% O_2_ in N_2_. Transfection of RBC infected with ring stage parasites with 100 µg of plasmid DNA was performed as previously described (Fidock and Wellems, 1997), and, the following day, drug selection with 2.5 nM WR99210 (Jacobus Pharmaceuticals) and/or blasticidin S hydrochloride (2 µg/mL; Sigma-Aldrich) was applied.

### Glucosamine knockdown of gene expression

Parasites were synchronized at the ring stage using 5% D-sorbitol (Chem-supply) and split into two equal cultures at 0.5% parasitemia in 4% hematocrit; one culture was treated with 2.5 mM D-(+)-glucosamine hydrochloride (GlcN) (Sigma-Aldrich) and the other vehicle control. Parasites were then cultured as normal for at least two cycles and observed every 6 h via Giemsa-stained blood smears.

### SYBR™ Green parasite growth assays

For 6-day growth assays, ring-stage synchronized parasites were seeded at 0.5% parasitemia and grown in 5 mL of culture at 4% hematocrit, with or without the addition of 2.5 mM GlcN to the culture media. Parasites were then grown for three subsequent cycles, with three separate aliquots of parasite culture removed every 48 h at the ring stage and frozen at −80°C. Every 48 h, either parasite media were replaced with fresh media or cultures were split 1/5 to ensure parasite survival. For 10-day growth assays, synchronized parasites at the ring stage were seeded into 96-well plates at 10,000 parasites per well in 100 µL of total culture at 4% hematocrit. Parasites were then cultured for a total of five cycles with or without the addition of 2.5 mM GlcN to the culture media. Culture media were replaced every 48 h with fresh media to ensure parasite survival. At the end of five cycles, culture plates were frozen overnight at −80°C. To perform the SYBR™ assay, cultures were thawed, and 30 µL was transferred to a 96-well plate. A volume of 30 µL of lysis buffer [20 mM Tris (pH 7.5), 5 mM Ethylenediaminetetraacetic acid (EDTA), 0.008% (w/v) saponin, and 0.08% (v/v) Triton X-100] containing SYBR™ Green I (0.2 µL/mL; Invitrogen) was added to the well before incubating in the dark for 1 h. Following incubation, fluorescence was read on a GloMax® Explorer Multimode microplate reader (Promega) at excitation and emission wavelengths of 485 nm and 528 nm, respectively. All assays were done in triplicate, and at least three individual biological experiments were performed. Either a two-tailed unpaired *t*-test or a one-way ANOVA was used to determine statistical significance, with data presented as the mean and error bars representative of the standard deviation.

### Immunofluorescence analysis

Thin smears of *P. falciparum* infected RBC were made on glass slides, and the cells were fixed for 2 min at −20°C with 90% (v/v) acetone and 10% (v/v) methanol. Samples were blocked for 30 min with 1% (w/v) bovine serum albumin (BSA) (Sigma-Aldrich)/Phosphate buffered saline (PBS) and then treated with the relevant primary antibody in 0.5% BSA/PBS for 1 h [rabbit anti-EXP2 (1:1,000) (Bullen et al., 2012); mouse and rat anti-HA (1:500, Sigma-Aldrich); rabbit anti-HSP101 (1:500) (de Koning-Ward et al., 2009)], rabbit anti-cMyc (1:500, Sigma), rabbit anti-GEXP07 (1:500, gift from Leann Tilley), rabbit anti-PTP2 (1:500, gift from Alan Cowman), and then the appropriate AlexaFluor 488– or AlexaFluor 568–conjugated secondary antibody (1:2,000; Life Technologies), with three 5-min washes with 1× PBS between each step. Samples were mounted using ProLong^TM^ Gold antifade reagent with 4′,6-diamidino-2-phenylindole (DAPI; Thermo Fisher Scientific) and incubated overnight at 37°C. Images were acquired with a Nikon Eclipse T*i*2 microscope using × 100 magnification under oil. Images were processed using a Fiji software (version 2.1.0/1.53c). To assess protein export, only cells containing one nucleus (DAPI signal) were counted. Parasites were determined to have successfully exported when GEXP07 or PTP2 signals showed five or more puncta in the erythrocyte cytoplasm (Maurer’s clefts), whereas intermediate export was defined as when GEXP07 and PTP2 signals were mostly observed within the parasite with less than five GEXP07 or PTP2 puncta in the erythrocyte cytoplasm. Conversely, no export was defined as GEXP07 and PTP2 puncta localizing within the parasite and lack of signal in the erythrocyte cytoplasm.

### Sequential solubility assay

Saponin-lysed trophozoite-stage pellets were gently washed three times in 500 µL of 1× PBS + protease inhibitors (PIs; Roche Holding AG). Pellets were then resuspended in hypotonic lysis buffer (1 mM HEPES, pH 7.4) and subjected to three rounds of snap freezing in liquid nitrogen and thawing. The solution was then incubated for 30 min on ice, mixing the solution every 10 min. A 100-µL aliquot of the resulting solution (total lysate) was taken and stored at −20°C before the remaining solution was centrifuged at 100,000 × g for 30 min at 4°C. The supernatant, containing soluble proteins, was collected, and the pellet was gently washed three times in 1× PBS + PI before being resuspended in 100 mM Na_2_CO_3_. After a 30-min incubation on ice, the solution was centrifuged (100,000 × g for 30 min at 4°C), and the supernatant, containing membrane-associated proteins, was collected. The pellet was washed further three times, then resuspended in 1% Triton X-100, and incubated for further 30 min on ice. The sample was then centrifuged (100,000 × g for 30 min at 4°C), and the supernatant containing integral membrane proteins was collected. The pellet containing insoluble proteins was finally washed three times resuspending in 1% Triton X-100. All samples were then separated using Sodium dodecyl-sulfate polyacrylamide gel electrophoresis (SDS-PAGE) and analyzed by Western blot, using the following antibodies: rabbit anti-Glyceraldehyde 3-phosphate dehydrogenase (GAPDH) (1:1,000), rabbit anti-HSP101 (1:1,000), rabbit anti-EXP2 (1:1,000), rabbit anti-cMyc (1:1,000), rabbit anti-hsp70 (1:1,000), and rabbit anti-SERA5 (1:1,000).

### Protein immunoprecipitation

A volume of ~20 µL of either rabbit anti–c-Myc agarose beads (Sigma-Aldrich), mouse-HA agarose beads (Sigma-Aldrich), or protein G agarose beads (Sigma-Aldrich) were equilibrated through three separate washes with 1% Triton X-100 in PBS + PI. The protein G beads were then conjugated to 20 µL of rabbit EXP2 antibody by incubating both together for a minimum of 1 h at 4°C with constant agitation. Parasites extracted with 0.1% saponin (Sigma-Aldrich) were lysed with 1% Triton X-100/PBS + PIs and following incubation for 1 h at 4°C, the lysate was centrifuged at 10,000 × *g* for 5 min at 4°C. The supernatant was then incubated with unconjugated protein G agarose beads for a minimum of 1 h at 4°C with constant agitation to pre-clear lysate of unspecific protein G binding. The lysate was then incubated with the equilibrated anti–c-Myc agarose or EXP2-conjugated protein G beads for 4 h at 4°C with constant agitation, and the beads were washed three times with 1% Triton X-100/PBS + PI followed by two washes in 1× PBS + PI. For Western blotting, precipitated proteins were eluted off the beads by incubating with pre-heated (60°C) 2× non-reducing sample buffer [1% sodium dodecyl sulfate (SDS), 10% glycerol, 1 mM EDTA, 0.005% bromophenol blue, and 50 mM Tris hydrochloride (pH 6.8)] for 5 min. Dithiothreitol was added to the eluted proteins at a final concentration of 50 mM.

### Quantitative reverse transcription polymerase chain reaction

To examine transcription of *exp2* in *Pf*EXP2-HA*glmS*/pRESA5′-EXP2-cMyc and *Pf*EXP2-HA*glmS*/pPTEX150 5′-EXP2-cMyc parasites, trophozoite-stage parasites were harvested by lysing infected RBC with 0.15% saponin in PBS. RNA was extracted from parasites using the RNeasy Mini Kit according to the manufacturer’s protocol (Qiagen). cDNA was synthesized using a QuantiTec cDNA synthesis kit (Qiagen) and then subjected to quantitative reverse-transcription PCR using 2× SensiFASTMix™ SYBR® Low-ROX master mix (Bioline) with the primers for endogenous *exp2* (DO1812/DO1813), *exp2* tagged with *cMyc* (DO1869/DO1870), and the housekeeping gene fructose-bisphosphate aldolase (*fbpa*, PF3D7_1444800) (DO1810/DO1811) as indicated in **Supplementary Table 1**. The expression level of *exp2* was normalized against the *fbpa* housekeeping gene, and the fold change was calculated using the 2^ΔΔCt^ method (Rao et al., 2013).

## Results

### Episomal EXP2 expression using the *hsp86* promoter can complement the loss of endogenous EXP2

EXP2 is essential to blood-stage development, and, thus, to examine whether variations of EXP2 or GRA17 could complement the function of endogenous EXP2, an inducible knockdown system was required. A *P. falciparum* EXP2-inducible knockdown line has previously been created, termed *Pf*EXP2-HA*glmS* (Charnaud et al., 2018). In this line, the 3′ untranslated region (UTR) of EXP2 has been replaced with a heterologous 3′ UTR containing the *glmS* ribozyme. And the EXP2 CDS has been fused at its C-terminus to a triple hemagglutinin (HA) tag (**Supplementary Figure 1A**). Inducible knockdown of EXP2 can be achieved through addition of glucosamine (GlcN) to the culture medium, which activates the ribozyme and results in cleavage of the poly-A tail, destabilization of the RNA transcript, and loss of EXP2 translation. Western blots of proteins extracted from schizont-stage *Pf*EXP2-HA*glmS* parasites grown in the presence of 2.5 mM GlcN revealed very efficient knockdown of EXP2 of > 99% (**Supplementary Figure 1B**).

**Figure 1:**
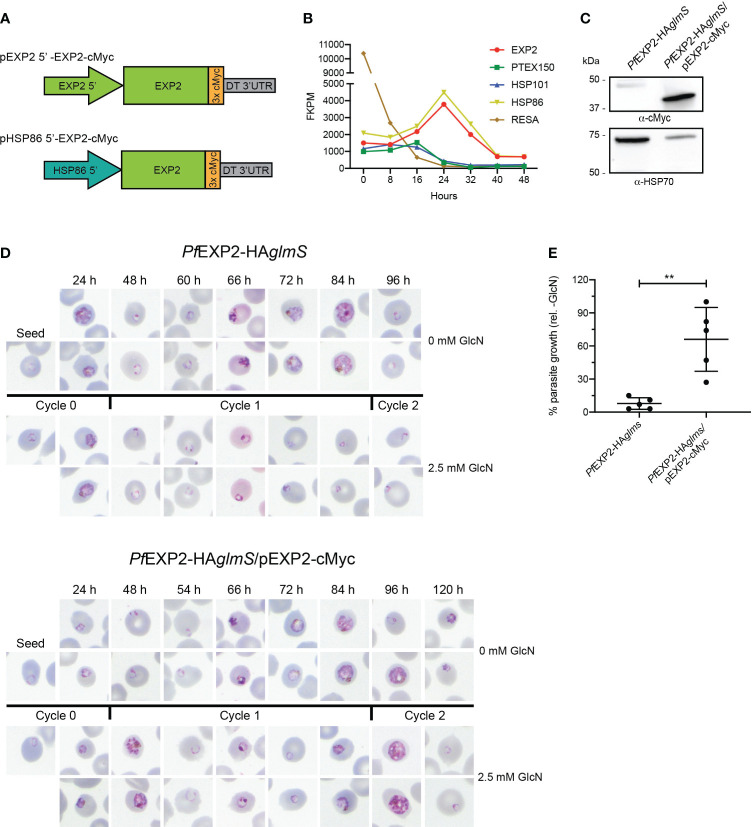
Complementation of *Pf*EXP2-HAglmS with EXP2-cMyc. **(A)** Schematic of constructs to drive expression of a C-terminally cMyc-tagged EXP2 via the *exp2* or *hsp86* promoter. **(B)** Transcription analysis of the indicated genes across a single erythrocytic cycle. Data extracted from Otto et al. (Otto et al., 2010). FPKM, fragments per kilobase of exon per million mapped fragments. **(C)** Western blot analysis of parasite lysates confirms that *Pf*EXP2-HA*glmS*/pEXP2-cMyc parasites express EXP2-cMyc (expected molecular weight is 39 kDa, with the 50-kDa band being non-specific labeling). HSP70 serves as a loading control. **(D)** Representative Giemsa-stained blood smears of three experiments where parasite lines were grown in the presence or absence of 2.5 mM GlcN after seeding at the stage indicated. **(E)** Growth of parasite lines after culturing for 10 days in the presence of 2.5 mM GlcN relative to untreated control as measured by Sybr Green 1 assay. Shown is the mean ± SD from five biological replicates, with significance determined by an unpaired *t-*test (**p < 0.01).

Before observing the effect that either modifying the structure of EXP2 or replacing with GRA17 has on parasite survival and growth, it first needed to be validated that endogenous EXP2 could be successfully complemented with *P. falciparum* wild-type EXP2 when expressed from episomes. However, no parasites could be recovered following transfection of the *Pf*EXP2-HA*glmS* parasite line with pEXP2 5′-EXP2-cMyc (**Figure 1A**) and selection with blasticidin-S or parasites that were recovered failed to express cMyc (data not shown). Thus, the endogenous *exp2* promoter was replaced with the *hsp86* promoter that shows a similar temporal expression pattern to *exp2* (**Figures 1A, B**) (Otto et al., 2010). A Western blot of lysate from *Pf*EXP2-HA*glmS* parasites transfected with pHSP86 5′-EXP2-cMyc (hereafter called *Pf*EXP2-HA*glmS*/EXP2-cMyc) and probed with a cMyc antibody revealed a band corresponding to the approximate size of cMyc-tagged EXP2 (39 kDa), indicating that this promoter could drive EXP2 expression (**Figure 1C**). Unless otherwise stated, all subsequent transgenes were expressed from the *hsp86* promoter.

Knockdown experiments were next performed on *Pf*EXP2-HA*glmS* and *Pf*EXP2-HA*glmS*/pEXP2-cMyc parasites by supplementing the growth medium with 2.5 mM GlcN at ring stage, with vehicle as a control. Smears taken from GlcN-treated *Pf*EXP2-HA*glmS*–infected RBC revealed that parasites stalled at the ring stage in the following cycle (cycle 1) before eventually condensing into small pyknotic forms, as previously observed (Charnaud et al., 2018). The *Pf*EXP2-HA*glmS*/pEXP2-cMyc line grew slower than *Pf*EXP2-HA*glmS* by ~12 h but, nevertheless, the episomal expression of EXP2-cMyc could rescue the growth of parasites when endogenous EXP2 expression was depleted, and parasites could complete cycle 1 (**Figure 1D**). Proliferation of both parasite lines was followed for 10 days via SYBR™ Green I assays (**Figure 1E**). Whereas growth of *Pf*EXP2-HA*glmS* treated with GlcN was reduced by 92% ± 5% by day 10, *Pf*EXP2-HA*glmS*/pEXP2-cMyc parasites cultured in the presence of GlcN were still able to proliferate. These experiments indicated that EXP2-cMyc expressed from the *hsp86* promoter is sufficient to enable parasite survival upon the depletion of endogenous EXP2.

### The EXP2 acidic C-terminal tail is essential for EXP2 function

**Figure 2:**
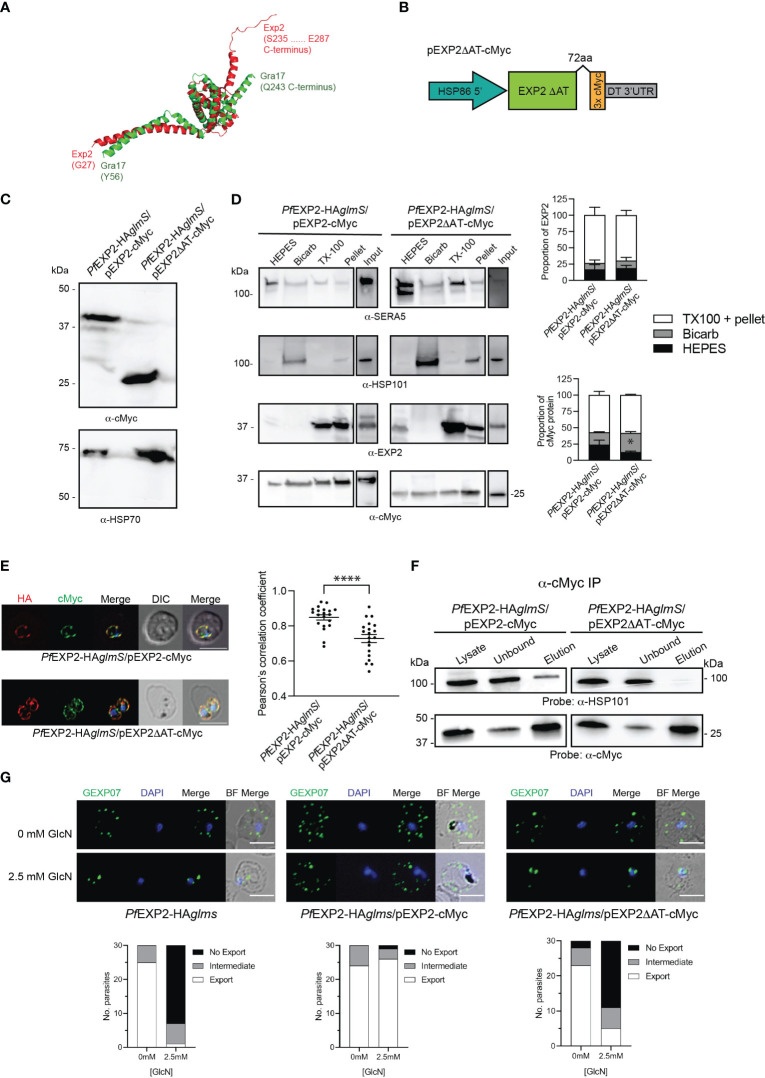
Complementation of *Pf*EXP2-HAglmS with episomally expressed truncated EXP2ΔAT-cMyc. **(A)** Pymol alignment of the EXP2 structure (pdb 6E10 chain B residues 27–234) and the structured region of GRA17 (residues 55–243) generated using Alphafold. **(B)** Schematic of plasmid construct used for complementation. **(C)** Western blot analysis of parasite lysates confirms EXP2 is truncated in *Pf*EXP2-HA*glmS*/EXP2ΔAT-cmyc (expected molecular weight is 30 kDa). **(D)** Representative Western blot from samples obtained from sequential solubility assays (left) and quantification of endogenous (upper graph) and cMyc-tagged protein (lower graph) in each fraction from three biological replicates. SERA5 is used as a marker for cytosolic proteins, HSP101 for membrane-associated proteins, and EXP2 for integral membrane proteins. Shown is the mean ± SEM from three biological replicates, with statistical significance determined by unpaired *t-*test (*p < 0.05). Note that input lanes were run at the same time but are shown as separate panels due to their location on the gel. **(E)** IFA of infected erythrocytes with DAPI staining (blue) marking the parasite’s nucleus (left), and the degree of co-localization between endogenous EXP2 (HA) and episomally expressed cMyc-tagged EXP2 calculated by measuring Pearson’s coefficients of merged Z-stack images of 20 cells (right). Shown is the mean ± SD, with significance determined by an unpaired *t-*test (****p < 0.001). **(F)** Western blots of whole-parasite lysate, unbound fraction and elution fraction following immunoprecipitation of cMyc-tagged proteins reveal that truncated EXP2 cannot interact with HSP101. **(G)** IFA of infected erythrocytes using antibodies to the Maurer’s cleft protein GEXP07 and quantification of GEXP07 localization (n = 30 cells from a single experiment), defined as either fully exported, not exported, or showing an intermediate export phenotype. Scale bar, 5 μm.

EXP2 and GRA17 share 23% sequence identity and 59% similarity; however, overlaying the tertiary structure of EXP2 (pdb 6E10; Ho et al., 2018) with a model of the structured region of GRA17 generated by AlphaFold (Jumper et al., 2021) revealed that the sequence pairwise alignment does not reflect the structural alignment where there appears to be a register shift in most of the helices and the C-terminus of GRA17 aligns with the linker helix of EXP2 (**Supplementary Figure 2**). Notably EXP2 extends beyond this region with an additional acidic C-terminal region, herein referred to as the acidic tail (AT; residues 216–287) (**Figure 2A**, **Supplementary Figure 2**), which we speculated enables EXP2 to function beyond a nutrient pore, enabling assembly with the other core PTEX components to permit protein export. To test the importance of the AT in assembly of EXP2 into the PTEX complex, *Pf*EXP2-HA*glmS* parasites were transfected with pEXP2_ΔAT_-cMyc, which encodes a truncated EXP2 lacking the AT (**Figure 2B**). Western blot analysis of *Pf*EXP2-HA*glmS*/pEXP2_ΔAT_-cMyc parasite lysate revealed that a cMyc-tagged protein of ~25 kDa was expressed, which is slightly smaller than the expected size of the C-terminal truncated EXP2 protein (30 kDa) (**Figure 2C**). A sequential solubility assay was performed on trophozoite-stage parasites to identify whether the removal of the AT affects the membrane binding of EXP2. There was no significant difference in the amount of total EXP2 in the soluble (HEPES) fraction, the membrane-associated (Bicarb) fraction, or the membrane-bound/insoluble material (TX-100 and Pellet) between the *Pf*EXP2-HA*glmS*/pEXP2-cMyc and *Pf*EXP2-HA*glmS*/pEXP2_ΔAT_-cMyc lines (**Figure 2D**, upper graph). When looking specifically at cMyc-tagged EXP2 (**Figure 2D**, lower graph), there was a small but significant increase in the amount of EXP2_ΔAT_-cMyc in the membrane-associated fraction, but no change to the amount of EXP2_ΔAT_-cMyc in the insoluble fraction relative to EXP2-cMyc, suggesting that EXP2_ΔAT_-cMyc can still be incorporated into the PVM. By Immunofluorescence analysis (IFA), both EXP2-HA and EXP2-cMyc co-localized and showed a high Pearson’s correlation coefficient (**Figure 2E**, top panel and graph). However, there was significantly less colocalization of EXP2-HA with EXP2_ΔAT_-cMyc, suggesting that removal of the C-terminal tail affects the localization of EXP2 at the PVM.

**Figure 3:**
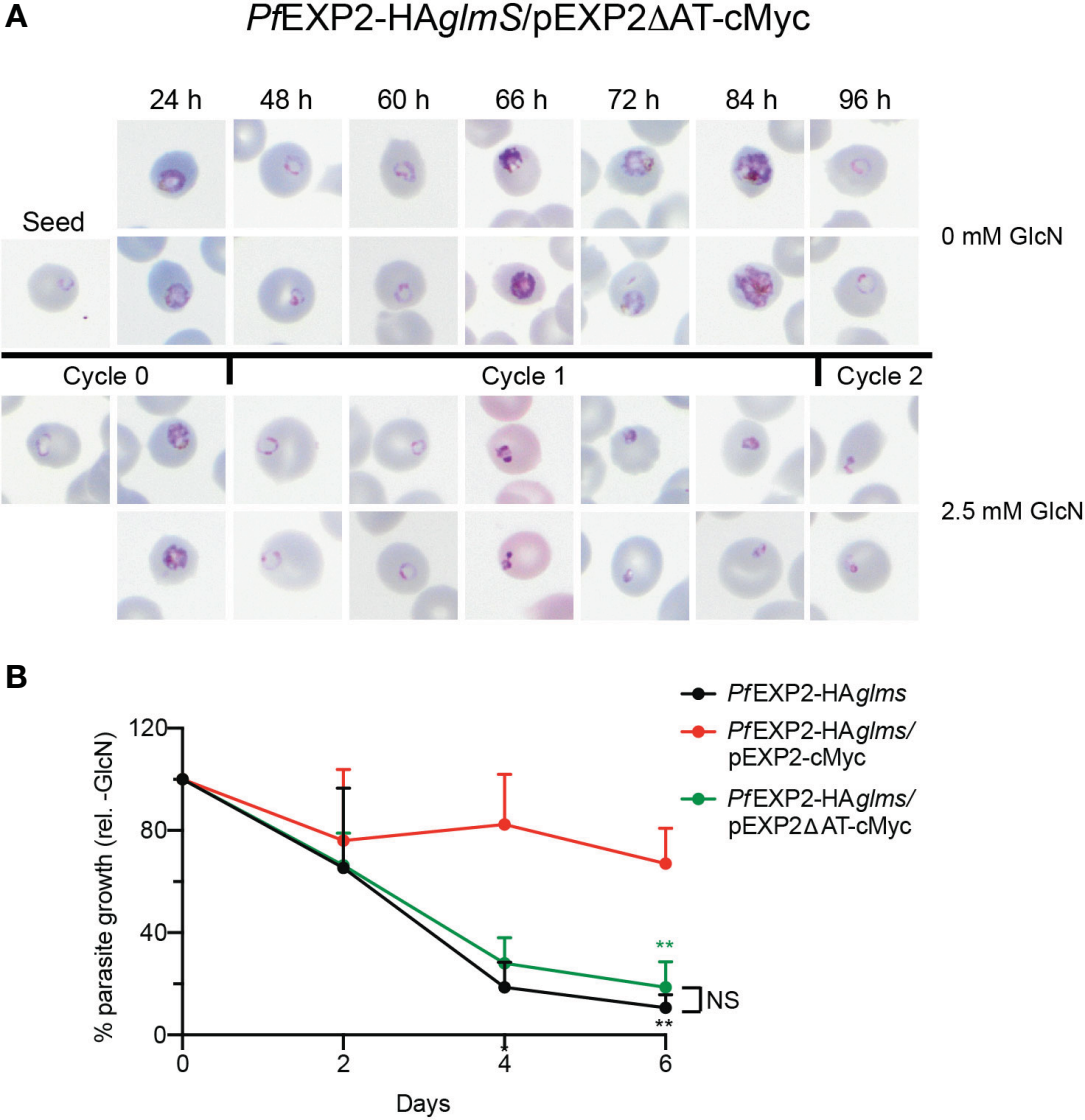
The acidic C-terminus of EXP2 is essential for parasite survival. **(A)** Representative Giemsa-stained blood smears of three experiments where *Pf*EXP2-HA*glmS*/pEXP2ΔAT-cMyc parasites were grown in the presence or absence of 2.5 mM GlcN after seeding at the stage indicated. **(B)** Growth of the indicated parasite lines in the presence of 2.5 mM GlcN relative to untreated control as measured by Sybr Green 1 assay. Shown is the mean ± SD from three biological replicates. A one-way ANOVA with Dunnett’s test for multiple comparisons was used to determine whether differences in growth were significant, with that relative to *Pf*EXP2-HA*glmS*/pEXP2-cMyc indicated (**p < 0.01); NS, not significant.

Immunoprecipitation (IP) of *Pf*EXP2-HA*glmS*/pEXP2-cMyc or *Pf*EXP2-HA*glmS*/pEXP2_ΔAT_-cMyc trophozoite-stage parasite lysate using cMyc antibodies followed by Western blotting was next performed to examine assembly of EXP2 into the PTEX complex. The initial lysate, unbound fraction, and IP eluate were all probed with antibodies recognizing the PTEX core constituent protein HSP101 (**Figure 2F**). EXP2-cMyc was able to pull down HSP101 as expected, whereas, in contrast, EXP2_ΔAT_-cMyc failed to pull down HSP101, indicating that the AT is required for EXP2 to assemble with HSP101 (**Figure 2F**). These results were confirmed by performing IFA experiments, and examining the ability of the Maurer’s cleft protein, gametocyte-exported protein 7 (GEXP07) (Sleebs et al., 2014; Mchugh et al., 2020), to be exported as proper assembly of EXP2 at the PVM and with the other core PTEX components is required for export. Both *Pf*EXP2-HA*glmS* parasites grown in the absence of GlcN and GlcN-treated *Pf*EXP2-HA*glmS*/EXP2-cMyc parasites could export this protein as indicated by the punctate labeling in the RBC cytoplasm (**Figure 2G**). In contrast, both GlcN-treated *Pf*EXP2-HA*glmS* and *Pf*EXP2-HA*glmS*/EXP2_ΔAT_-cMyc parasites failed to export GEXP07 (**Figure 2G**). In keeping with these results, *Pf*EXP2-HA*glmS*/EXP2_ΔAT_-cMyc parasites failed to make it past the ring stage in the cycle following knockdown (**Figure 3A**), mimicking the parasite death phenotype seen for GlcN-treated *Pf*EXP2-HA*glmS* parasites (**Figure 1D**). Growth of *Pf*EXP2-HA*glmS*/pEXP2_ΔAT_-cMyc parasites was also analyzed by performing SYBR™ Green assays, which demonstrated that most parasites had died (81% ± 10%) by day 6, which is comparable to treated *Pf*EXP2-HA*glmS* parasites (89% ± 5%). In contrast and in keeping with the previous experiments, *Pf*EXP2-HA*glmS*/pEXP2-cMyc parasites expressing full-length EXP2-cMyc could survive following knockdown of endogenous EXP2 (67% ± 14% survival) (**Figure 3B**). Together, these assays indicate that truncation of the EXP2 AT is detrimental to the survival of *P. falciparum*.

### GRA17 is unable to complement the function of EXP2

**Figure 4:**
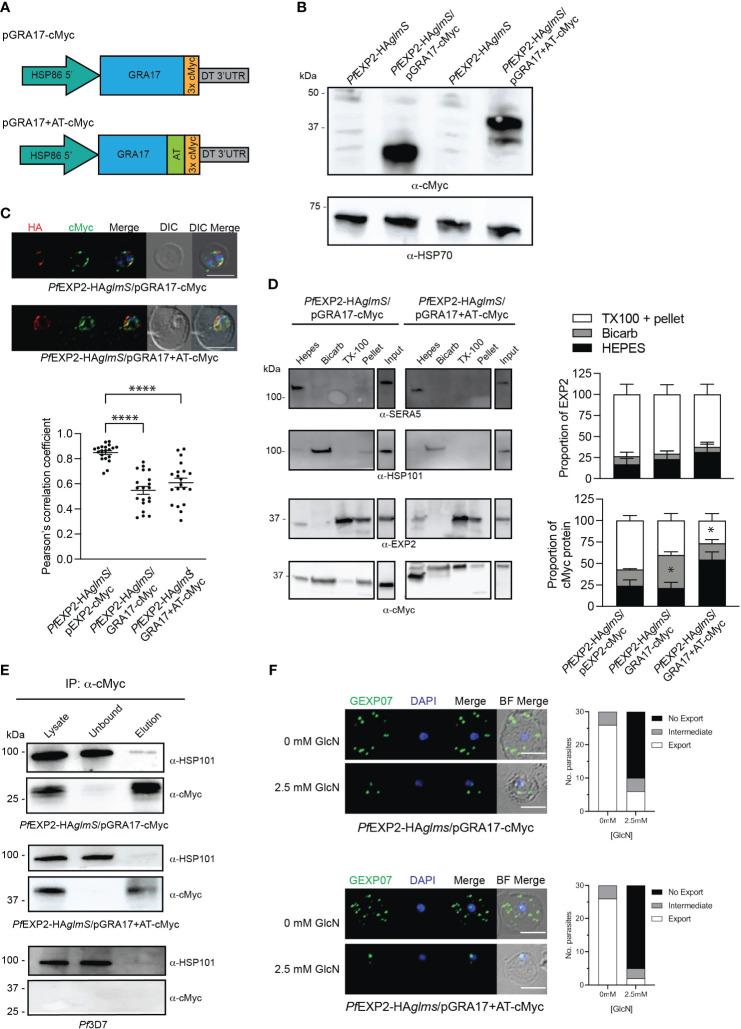
GRA17 with or without an acidic tail is unable to complement EXP2 function. **(A)** Schematic of GRA17 plasmid constructs used for complementation. **(B)** Western blot analysis of parasite lysates to confirm expression of GRA17-cMyc fusion proteins (expected molecular weight for GRA17-cMyc and GRA17-cMyc+AT is 31 and 40 kDa, respectively. **(C)** IFA of infected erythrocytes with DAPI staining (blue) marking the parasite’s nucleus (top), and the degree of co-localization between endogenous EXP2 (HA) and episomally expressed cMyc-tagged GRA17 calculated by measuring Pearson’s coefficients of merged Z-stack images of 20 cells from three experiments (bottom). Statistical significances were determined using one-way ANOVA with Welch correction and Dunnett’s test for multiple comparison (****p < 0.0001). **(D)** Representative Western blot of samples obtained from sequential solubility assays (left) and quantification of EXP2 (upper graph) and cMyc-tagged protein (lower graph) in each fraction from three biological replicates. SERA5 was used as a marker for cytosolic proteins, HSP101 for membrane-associated proteins, and EXP2 for integral membrane proteins. Shown is the mean ± SEM from three biological replicates, with statistical significance determined by unpaired *t-*test (*p < 0.05). Note that input lanes were run at the same time but are shown as separate panels due to their location on the gel **(E)** Western blots (n = 3) of whole-parasite lysate, unbound fraction and elution fraction following immunoprecipitation of cMyc-tagged proteins reveal that GRA17-cMyc proteins do not interact with HSP101. Lysates from *Pf*3D7 serve as a negative control. **(F)** IFA of infected erythrocytes using antibodies to the Maurer’s cleft protein GEXP07 and quantification of GEXP07 localization (n = 30 cells from a single experiment), defined as either fully exported, not exported, or showing an intermediate export phenotype. Scale bar, 5 μm.

EXP2 has been shown to complement the function of GRA17 (Gold et al., 2015). However, it has not been determined whether the converse is possible, and, if the pore-forming capacity of GRA17 can complement the function of both roles that EXP2 serves in *P. falciparum* or whether the lack of an AT would prevent assembly of GRA17 into the PTEX complex. To examine this, two constructs were transfected into *Pf*EXP2-HA*glmS* parasites; one that encodes GRA17 (pGRA17-cMyc) and the other in which the AT of EXP2 is fused in frame to the C-terminus of GRA17 (pGRA17+AT-cMyc; **Figure 4A**). Transfectants were recovered on both occasions, and Western blot analysis of parasite lysates shows that *Pf*EXP2-HA*glmS*/pGRA17-cMyc and *Pf*EXP2-HA*glmS*/pGRA17+AT-cMyc express a cMyc-tagged protein that corresponds with the expected size of GRA17 incorporating a 3× cMyc tag alone (31 kDa) or both the EXP2 AT and 3× cMyc tag (40 kDa), respectively (**Figure 4B**). IFA showed that GRA17-cMyc and GRA17 + AT-cMyc mostly concentrated at the parasite periphery (**Figure 4C**). However, with both GRA17 lines, there was significantly less colocalization of cMyc with endogenous EXP2 (HA) when compared with the EXP2-cMyc complemented control line demonstrated by significantly lower Pearson’s correlation coefficient (**Figure 4C**, graph). Sequential solubility assays revealed that a proportion of GRA17-cMyc was present in the Triton X-100 pellets; however, there was also significantly more GRA17-cMyc in the membrane-associated fraction (Bicarb) when compared to EXP2-cMyc (**Figure 4D**). Unexepectedly, addition of the EXP2 AT to GRA17 affected the solubility profile of GRA17+AT-cMyc such that it was mostly found within the soluble fraction (HEPES), and, in one of the replicate experiments (shown here), the major proteins species detected in the soluble fraction were at a lower than expected molecular weight (**Figure 4D**, lower panel). Together, these results indicate that GRA17-cMyc is capable of both trafficking and inserting (albeit less efficiently) into the PVM, in keeping with the properties expected for an EXP2 ortholog, but that addition of the EXP2 AT hindered the ability of GRA17 to be incorporated into the membrane.

**Figure 5:**
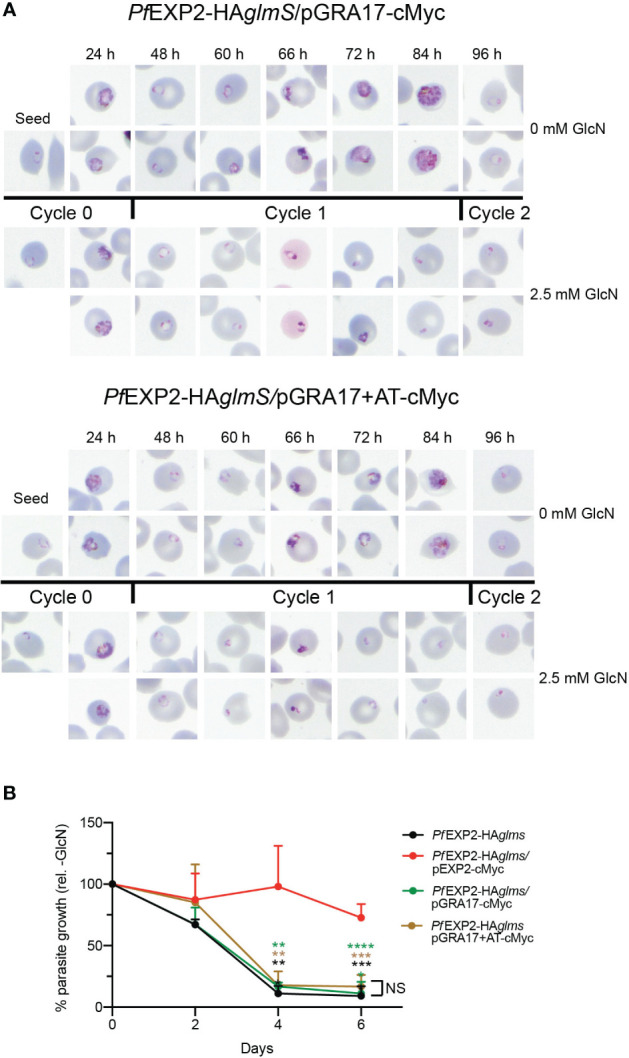
GRA17 cannot complement the function of EXP2 even with addition of the EXP2 acidic tail. **(A)** Representative Giemsa-stained blood smears of three experiments where parasites were grown in the presence or absence of 2.5 mM GlcN after seeding at the stage indicated. **(B)** Growth of the indicated parasite lines in the presence of 2.5 mM GlcN relative to untreated control. Shown is the mean ± SD from three biological replicates. One-way ANOVA with Dunnett’s test for multiple comparisons was used to determine whether differences in growth were significant, with that relative to *Pf*EXP2-HA*glmS*/pEXP2-cMyc indicated (**p < 0.01; ***p < 0.001; ****p < 0.0001); NS, not significant.

IP experiments using anti-cMyc revealed that both GRA17-cMyc and GRA17+AT-cMyc showed a very weak association with HSP101 (**Figure 4E**), which is most likely to be non-specific binding given that there is also a weak band in the elution fraction of the negative control when lysate from *Pf*3D7 wild-type parasites was used in the IP. This indicates that neither GRA17-cMyc nor GRA17+AT-cMyc is assembling as a pore-forming component of PTEX, and, in keeping with this, when *Pf*EXP2-HA*glmS*/GRA17-cMyc and *Pf*EXP2-HA*glmS*/GRA17+AT-cMyc were treated with GlcN, they failed to export GEXP07 (**Figure 4F**). Moreover, both parasites stalled at the ring stage in the cycle following the addition of GlcN, leading to parasite death in an analogous fashion to *Pf*EXP2-HA*glmS* parasites (**Figure 5A**), with only 11% ± 10% and 17% ± 10% of *Pf*EXP2-HA*glmS*/GRA17-cMyc and *Pf*EXP2-HA*glmS*/GRA17-AT-cMyc parasites, respectively, surviving relative to no GlcN control after 6 days in culture (**Figure 5B**). These combined data indicate that EXP2 is a unique pore and its assembly into PTEX and resulting protein export function cannot be complemented by a homologous pore protein. Moreover, despite the AT of EXP2 being essential for its assembly into PTEX, this sequence is not sufficient to allow an orthologous pore protein to assemble into the PTEX complex.

### Altering the temporal expression of EXP2 via promoter switching

In the final stages of the *P. falciparum* erythrocytic cycle, the PTEX components are packaged into the dense granules of the daughter merozoites. This process is essential for effective release of the PTEX components into the PV during invasion, therefore allowing PTEX to be assembled and inserted into the PVM and protein export to occur during the ring stage of development (de Koning-Ward et al., 2009; Bullen et al., 2012). This process is in keeping with the peak expression profiles for *ptex150* and *hsp101*. However, the *exp2* expression profile differs from that of *ptex150* and *hsp101*, instead peaking at 24 h which coincides with the trophozoite stage of development (**Figure 1B**). As EXP2 also serves as a nutrient pore (Garten et al., 2018), it is likely that the different timing of EXP2 expression allows it to also assist with nutrient transfer to sustain the rapid growth that occurs during the trophozoite stage. Therefore, it was of interest to see what would happen if the timing of *exp2* expression was altered to mirror that of the other PTEX components and if it was detrimental to the growth of the parasite.

**Figure 6:**
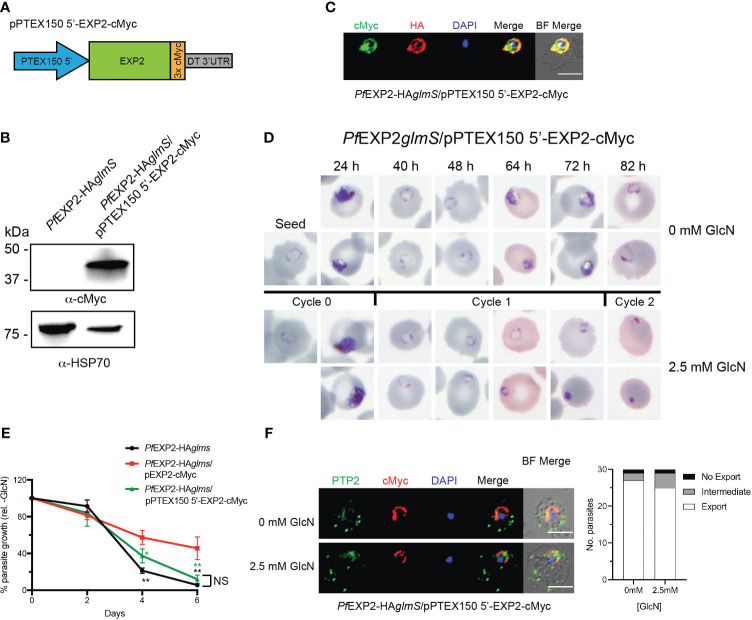
Expression of EXP2 under the *ptex150* promoter affects parasite growth but not protein export. **(A)** Schematic of construct to drive expression of a C-terminally cMyc-tagged EXP2 via the *ptex150* promoter. **(B)** Western blot analysis of parasite lysate to confirm expression of EXP2-cMyc (expected molecular weight is 39 kDa). **(C)** Representative IFA (n = 3) of infected erythrocytes with DAPI staining (blue) marking the parasite’s nucleus shows that endogenous EXP2 (HA) and cMyc-tagged EXP2 co-localize. **(D)** Representative Giemsa-stained blood smears of three experiments in which parasites were grown in the presence or absence of 2.5 mM GlcN. **(E)** Growth of the indicated parasite lines in the presence of 2.5 mM GlcN relative to untreated control. Shown is the mean ± SD from three biological replicates. One-way ANOVA with Dunnett’s test for multiple comparisons was used to determine whether differences in growth were significant, with that indicated relative to *Pf*EXP2-HA*glmS*/pEXP2-cMyc (*p < 0.05; **p < 0.01); NS, not significant. **(F)** IFA of *Pf*EXP2-HA*glmS*/pPTEX150 5′-EXP2-cMyc–infected erythrocytes using antibodies to the Maurer’s cleft protein PTP2 and quantification of PTP2 localization (n = 30 cells from a single experiment), defined as either fully exported, not exported, or showing an intermediate export phenotype. Scale bar, 5 μm.

Initially, the *ptex150* promoter was used in place of the *hsp 86* promoter to drive expression of EXP2. Lysates of *Pf*EXP2-HA*glmS* parasites transfected with pPTEX150 5′-EXP2-cMyc (**Figure 6A**) were shown to express a protein of the expected size for cMyc-tagged EXP2 (39 kDa) (**Figure 6B**). IFA validated cMyc expression and localization of EXP-cMyc to the PVM (**Figure 6C**). Examination of Giemsa-stained smears of *Pf*EXP2-HA*glmS*/pPTEX150 5′-EXP2-cMyc following knockdown of endogenous EXP2 revealed that parasites were dying in the cycle following GlcN treatment, such that parasites could not progress beyond the ring stage (**Figure 6D**). After 6 days in culture, following knockdown, there was a 90% ± 5% reduction in parasite growth (**Figure 6E**). From these data, it can be inferred that alteration of the expression profile of *exp2* to mimic that of the core PTEX component PTEX150 is detrimental to the parasite’s survival. To further investigate the observed phenotype, the ability of *Pf*EXP2-HA*glmS*/pPTEX150 5′-EXP2-cMyc to export cargo via PTEX was tested. Parasites were harvested prior to the onset of the growth defect and tested for their ability to export the electron dense vesicle protein *P. falciparum* erythrocyte membrane protein 1 trafficking protein 2 (*Pf*PTP2) (Regev-Rudzki et al., 2013; Rug et al., 2014) as antibodies to GEXP07 were no longer available. Unexpectedly, *Pf*EXP2-HA*glmS*/pPTEX150 5′-EXP2-cMyc parasites treated with GlcN were able to effectively export *Pf*PTP2 (**Figure 6F**), suggesting that the cause of parasite death following the alteration of EXP2 expression is independent of PTEX formation.

**Figure 7:**
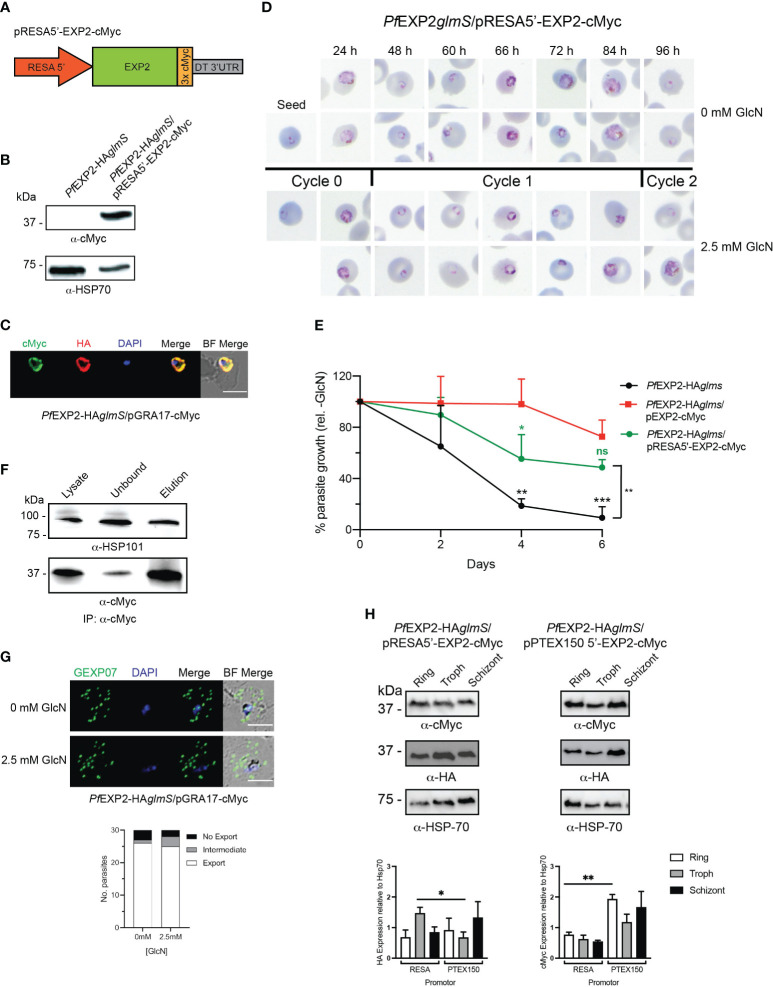
Expression of EXP2 under the *resa150* promoter. **(A)** Schematic of construct to drive expression of a C-terminally cMyc-tagged EXP2 via the *resa* promoter. **(B)** Western blot analysis of parasite lysate to confirm expression of EXP2-cMyc (expected molecular weight is 39 kDa). **(C)** Representative IFA (n = 3) of infected erythrocytes with DAPI staining (blue) marking the parasite’s nucleus (left) shows that endogenous EXP2 (HA) and cMyc-tagged EXP2 co-localize. **(D)** Representative Giemsa-stained blood smears of three experiments where parasites were grown in the presence or absence of 2.5 mM GlcN. **(E)** Growth of the indicated parasite lines in the presence of 2.5 mM GlcN relative to untreated control. Shown is the mean ± SD from three biological replicates. One-way ANOVA with Dunnett’s test for multiple comparisons was used to determine whether differences in growth were significant, with that relative to *Pf*EXP2-HA*glmS*/pEXP2-cMyc indicated (*p < 0.05; **p < 0.01; ***p < 0.001; ns, not significant). **(F)** Western blots (n = 3) of whole-parasite lysate, unbound fraction and elution fraction following immunoprecipitation of cMyc-tagged protein shows that EXP2-cMyc interacts with HSP101. **(G)** IFA of infected erythrocytes using antibodies to the Maurer’s cleft protein GEXP07 and quantification of GEXP07 localization (n = 30 cells from a single experiment), defined as either fully exported, not exported, or showing an intermediate export phenotype. Scale bar, 5 μm. **(H)** Western blots of whole-parasite lysate from the indicated lines and quantitation of EXP2 (detected via anti-HA antibody) or episomally expressed EXP2 (detected anti-cMyc antibody) when expressed from either the *resa* or *ptex150* promoter. Shown is the mean ± SEM from three biological replicates, with statistical significance determined by unpaired *t-*test (*p < 0.05; **p < 0.01).

Because *P. falciparum* blood-stage transcriptome analysis indicates that *ptex150* expression is weaker than *exp2* (Otto et al., 2010), we replaced the *hsp86* promoter with that of *resa* because this protein is abundantly expressed in parasites. Similarly to the PTEX components, ring-infected erythrocyte surface antigen (RESA) is trafficked to the dense granules during schizont maturation, where upon merozoite invasion, it is secreted into the PV and then exported by the assembled PTEX complex (Brown et al., 1985; Aikawa et al., 1990; Culvenor et al., 1991; Riglar et al., 2011; Riglar et al., 2013). Lysates of PfEXP2-HA*glmS* transfected with pRESA5′-EXP2-cMyc (**Figure 7A**) expressed a cMyc protein of similar size to that expected if EXP2 had been successfully tagged with cMyc (39 kDa) (**Figure 7B**). IFA of trophozoite-stage parasites confirmed cMyc expression and demonstrated that, despite being placed under the expression of the *resa* promoter, EXP2-cMyc co-localized with endogenous EXP2 at the PVM (**Figure 7C**).

Knockdown of the endogenous EXP2 and examination of Giemsa-stained smears revealed a growth delay of *Pf*EXP2-HA*glmS*/pRESA5′-EXP2-cMyc in the cycle following GlcN treatment (**Figures 7D, E**). Notably, *Pf*EXP2-HA*glmS*/RESA5′-EXP2-cMyc parasites were able to complete cycle 1 and reinvade new RBCs following GlcN treatment (**Figure 7D**), and, although the parasitemia was lower than *Pf*EXP2-HA*glmS*/pEXP2-cMyc at day 6, the difference was not statistically significant (**Figure 7E**). Likewise, a 10-day growth assay also confirmed that there were no significant differences in the ability of EXP2 expressed off the *hsp86* promoter or *resa* promoter to complement endogenous EXP2 (data not shown). Thus, altering the expression profile of EXP2 by placing this protein under the control of the *resa* promoter is not detrimental to parasite growth. IP of EXP2-cMyc from *Pf*EXP2-HA*glmS*/pRESA5′-EXP2-cMyc parasite lysate using cMyc antibodies revealed that there is no adverse effect of altering the expression of EXP2 with respect to its ability to associate with HSP101 and assemble into PTEX (**Figure 7F**). In addition, GlcN-treated *Pf*EXP2-HA*glmS*/pRESA5′-EXP2-cMyc parasites were also able to efficiently export GEXP07 (**Figure 7G**), in keeping with a functional PTEX complex.

Because of the dual role of EXP2 as a nutrient pore and as a core constituent of PTEX, we hypothesized that the difference in growth phenotypes between *Pf*EXP2-HA*glmS*/pRESA5′-EXP2-cMyc and *Pf*EXP2-HA*glmS*/pPTEX150 stemmed from differences in the strength of the promotor, such that EXP2-cMyc expression under the *ptex150* promoter was insufficient for nutrient pore formation. To investigate, the relative abundance of cMyc-tagged EXP2 protein (**Figure 7H**) and mRNA (**Supplementary Table 2**) at each life cycle stage was compared between both lines. Unexpectedly, there was a significantly higher amount of endogenous EXP2-HA in *Pf*EXP2-HA*glmS*/pRESA5′-EXP2-cMyc at trophozoite stage when compared with that in *Pf*EXP2-HA*glmS*/pPTEX150 (**Figure 7H**, left graph), yet there were significantly higher amounts of EXP2-cMyc protein and transcript abundance at ring stages when expressed off the *ptex150* promotor (**Figure 7H**, right graph). Thus, we were unable to attribute the death phenotype to a decreased available pool of EXP2 for nutrient pore activity.

## Discussion

PTEX is unique to *Plasmodium*; however, homologs to EXP2 exist within other Apicomplexa, specifically those that replicate within a PV (Gold et al., 2015). One of these homologs is the *T. gondii* protein GRA17, a pore-forming protein that enables small-molecule transfer across the parasites PVM, but unlike EXP2, does not play a role in protein export. Nevertheless, EXP2 can successfully complement the function of GRA17 within *T. gondii* (Gold et al., 2015), and, indeed, EXP2 has been shown to perform dual roles during the asexual stage of *Plasmodium* development, serving as the pore-forming component of PTEX to facilitate protein export and in nutrient acquisition, where it acts independently of PTEX (Garten et al., 2018). Herein, GRA17 was probed for its ability to complement EXP2 function in *P. falciparum* to understand how EXP2 could potentially perform two roles. This study revealed that GRA17 was incapable of replacing EXP2 function as it was unable to assemble with the PTEX complex to serve as the protein export pore, resulting in a failure to export proteins and consequently parasites died. This indicates that EXP2 possesses unique features that are absent from a similar pore, which makes it specifically primed for PTEX association.

Alignment of EXP2 and GRA17 revealed that EXP2 has a unique acidic C-terminus, or tail, and complementation experiments with a truncated EXP2 revealed that the AT was essential for EXP2 to associate with HSP101 and, hence, to form the translocon to facilitate protein export. However, removal of the AT did not affect the membrane binding affinity of EXP2 in *Pf*EXP2_ΔAT_-cMyc parasites, indicating that interaction with HSP101 is not required for EXP2 insertion into the membrane. This is consistent with EXP2 being able to form nutrient pores independently of PTEX, as well as the reduced co-localization of EXP2_ΔAT_-cMyc with endogenous EXP2 that can form both types of pores at the PVM. Nevertheless, it is interesting to note that EXP2-cMyc, EXP2_ΔAT_-cMyc, and GRA17-cMyc, which were expressed from the *hsp86* promoter, could also be detected in the soluble fraction in addition to the Triton X-100 fractions, unlike endogenous EXP2 that was predominantly present in the Triton X-100 fractions. It remains unknown what drives the EXP2 insertion process that results in the formation of pores in the PVM and whether this relates to timing of expression or other factors but presumably this process is regulated so that EXP2 is not prematurely inserted into the dense granule membrane where it is stored prior to invasion (Bullen et al., 2012). In *T. gondii*, correct localization of GRA17 at the PVM requires additional GRA proteins, namely, GRA45, GRA70, and GRA72 (Paredes-Santos et al., 2023), and their absence from *P. falciparum* may explain the reduced efficiency of GRA17 insertion into the PVM, which, in turn, may lead to reduced interaction with HSP101. In the gene model suggested by Gold et al. (Gold et al., 2015), removal of the signal peptide would leave 31-residue N-terminal to the membrane-spanning helix, whereas, in comparison, EXP2 only has two residues, and this region in GRA17 may be important for correct localization. It is interesting to note that, despite EXP2 lacking this region, EXP2 can still be inserted into the PVM and form a pore in *T. gondii.* Before we began this study, the structural assembly of PTEX had been determined by cryo-EM (Ho et al., 2018). Within the complex, the assembly strand (D231–S234) of EXP2 projects upward to form contacts with HSP101. The C-terminus of EXP2 (S234–E287) was not visualized in the structure; however a previous study that truncated this highly charged 54–amino acid C-terminus showed that it was not essential to PTEX function and asexual parasite survival although parasites did exhibit a PVM channel with an altered voltage response (Garten et al., 2018). The EXP2 72–amino acid C-terminal AT truncation described herein (S216–E287) projects beyond the assembly strand and into a structure identified as the linker helix (L203–L231), therefore providing biochemical evidence that the assembly strand and linker helix are essential for correct EXP2 assembly into the PTEX complex. This is also in agreement with the study by Ho et al. (2018) who found that knockdown of EXP2 could not be rescued by a mutant of EXP2 that lacked the last 66 residues (N222–E287) (Ho et al., 2018).

Despite the importance of the AT for assembly of EXP2 into the PTEX complex, appendage of this sequence to GRA17 did not facilitate GRA17+AT incorporation into PTEX and was even detrimental to its ability to insert into the PVM, hence resulting in reduced co-localization with EXP2. This resulted in a failure to export proteins when endogenous EXP2 was depleted, indicating that additional sequences within EXP2 may be critical for key interactions with PTEX and its unique protein export pore function. Previously, GRA17 has been modeled in a heptamer formation (Paredes-Santos et al., 2023), and, therefore, we could align GRA17 with the known structure of EXP2 (Ho et al., 2018). Although overall their similarities are notable, there are several key differences that might provide some insights into why GRA17 alone or with the EXP2 AT could not complement EXP2 in *P. falciparum* parasites. The N-terminal transmembrane domain of GRA17 has a kink at Ser88 to accommodate a longer helix compared to that of EXP2, although the measurement of the membrane pore suggested that GRA17 and EXP2 were of similar size (Paredes-Santos et al., 2023). The EXP2 body domain consists of five helices B1–B5 that align relatively well between the two proteins; however, there are discrepancies in the length of helices and angular positions, which would arguably change the intricate protein–protein interactions formed with the interlacing PTEX150 monomers in the PTEX assembly. Interestingly, EXP2 contains a disulfide bond linking helix B2 and B3 to stabilize the body domain. GRA17 does not contain any cysteine residues, and this may affect the stability of GRA17 and its ability to assemble with the other PTEX components. Directly after, the body domain is a linker helix that faces vertical to place the remaining extension of EXP2 in a position to interact with HSP101 (Ho et al., 2018). In GRA17, the linker helix is not a linker but instead is the C-terminus and is modeled facing out onto the surface of the heptamer. In this position, it would be unable to form any interactions with HSP101 in the PTEX assembly. By attaching the AT of EXP2 onto the C-terminus of GRA17 would invariably extend the protein, this addition was still insufficient to complement EXP2 *in vivo*.

This study also explored the importance of the timing of *exp2* expression for *P. falciparum* survival, which normally peaks during the trophozoite stage of the parasite, compared to *hsp101* and *ptex150* that peak at the late schizont/ring stages of development to facilitate protein export from the ring stages. The unique transcriptional profile of *exp2* is thought to assist the parasite during its rapid growth period through the creation of EXP2 nutrient pores. Expression of EXP2 under the *ptex150* promoter failed to rescue EXP2 knockdown parasites and parasites died around the ring-trophozoite transition. In this case, PTEX was still functional as protein export was observed, suggesting that the cause of parasite death was likely to be the result of insufficient EXP2 pores to allow for nutrient influx during the rapid growth trophozoite stage and provides evidence that the nutrient channel activity of EXP2 is also essential to the parasite. This finding is in keeping with two recent studies that dissected the role of EXP1, an interacting partner of the nutrient pore form of EXP2 (Mesén-Ramírez et al., 2019; Nessel et al., 2020). Although the removal of EXP1 did not influence the ability of the parasite to export effector proteins, it did result in a loss of PVM solute channel activity and affected asexual development of *P. falciparum* parasites *in vitro*, indicating that the nutrient channel role of EXP2 is essential to the parasite. Conversely, we found that EXP2 knockdown could be rescued with EXP2-cMyc expressed off the *resa* promoter. Although the expression of endogenous EXP2 was higher at the trophozoite stage in *Pf*EXP2-HA*glmS*/pRESA5′-EXP2-cMyc compared with that in *Pf*EXP2-HA*glmS*/p150 5′-EXP2-cMyc in the absence of GlcN and coincides with when nutrient exchange is occurring, the knockdown of endogenous EXP2 is very efficient with GlcN, and, thus, this pool of EXP2 should have been depleted. Instead, EXP2 protein and mRNA levels of EXP2-myc were lower under the *resa* promoter when compared to the *ptex150* promoter at all stages of parasite development, demonstrating that promoters in episomes cannot tightly regulate gene expression like the endogenous locus. Thus, although the mechanism behind why the *resa* promoter but not the *ptex150* promoter can support EXP2 function remains to be resolved, it appears that EXP2 expression is finely tuned in the parasite to support its dual functions.

## Data availability statement

The raw data supporting the conclusions of this article will be made available by the authors, without undue reservation.

## Author contributions

EP: Formal Analysis, Writing – original draft, Writing – review & editing, Data curation, Investigation, Methodology. NC: Data curation, Formal Analysis, Investigation, Methodology, Writing – review & editing. JM: Data curation, Formal Analysis, Investigation, Methodology, Writing – review & editing. MC: Investigation, Writing – review & editing. PG: Writing – review & editing, Resources. CW: Writing – review & editing, Formal Analysis, Investigation. TdK-W: Formal Analysis, Writing – review & editing, Conceptualization, Funding acquisition, Project administration, Resources, Supervision, Validation, Writing – original draft.

## Supplementary material

Supplementary Figure 1 Inducible knockdown of EXP2. **(A)** Overview demonstrating how insertion of the *glmS* riboswitch into the *exp2* locus can lead to the conditional depletion of *exp2* with glucosamine (GlcN). **(B)** Left panel: Representative Western blot of *Pf*EXP2-HA*glms* protein lysates prepared from parasites treated with either 2.5 mM GlcN (+) or left untreated (-). HSP70 serves as the loading control. Right panel: Densitometry of bands observed in Western blots was performed using ImageJ to calculate the ratio of EXP2 expression in GlcN-treated parasites relative to HSP70 when compared to untreated parasites. Shown is the mean ± SD (n=2). Statistical significance was determined using an unpaired *t-*test. ****, *p*<0.001.

Supplementary Figure 2 Comparison between EXP2 and GRA17. **(A)** Coding sequence of EXP2, with the underlined sequence indicating the acidic tail (216-287). **(B)** Pairwise sequence alignment of GRA17 (56-243) and EXP2 (27-287) highlighting in bold the secondary structure elements. The underlined sequence represents the Acidic Tail of EXP2 (216-287) used in our experimental design. (B) AlphaFold model of GRA17 (56-243) in a heptamer assembly (grey)(Paredes-Santos et al., 2023). GRA17 monomer (green) aligned with a monomer of EXP2 (pdb 610, chain B, red). The model ends at EXP2 S235, so most of the AT is absent from this model. Alignment and schematic were generated in Pymol.

Supplementary Table 1 Oligonucleotides used in this study.

Supplementary Table 2 The fold changes of *exp2-cMyc* expression under the *ptex150* promoter relative to the *resa* promoter.
