# Supplementary figures and images for: Dissecting EXP2 sequence requirements for protein export in malaria parasites
Source: Front Cell Infect Microbiol. 2024 Jan 12;13:1332146. doi: 10.3389/fcimb.2023.1332146 (PMC10811066; doi:10.3389/fcimb.2023.1332146)

A

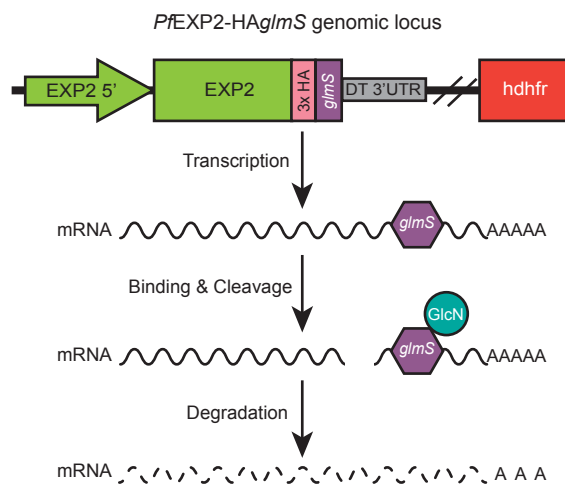

B

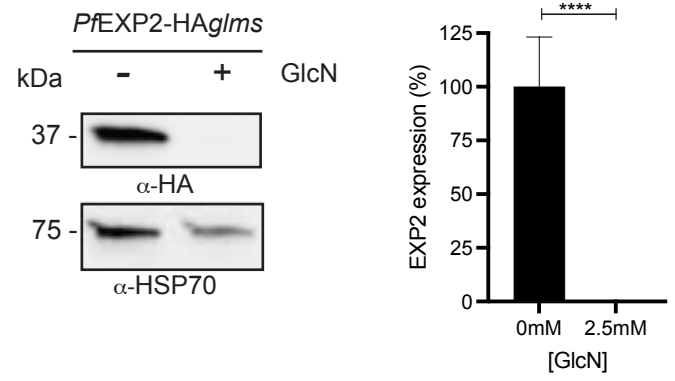

Supplement: Supplementary Figure 1 — Inducible knockdown of EXP2. (A) Overview demonstrating how insertion of the glmS riboswitch into the exp2 locus can lead to the conditional depletion of exp2 with glucosamine (GlcN). (B) Left panel: Representative Western blot of PfEXP2-HAglms protein lysates prepared from parasites treated with either 2.5 mM GlcN (+) or left untreated (-). HSP70 serves as the loading control. Right panel: Densitometry of bands observed in Western blots was performed using ImageJ to calculate the ratio of EXP2 expression in GlcN-treated parasites relative to HSP70 when compared to untreated parasites. Shown is the mean ± SD (n=2). Statistical significance was determined using an unpaired t-test. ****, p<0.001. [file DataSheet_1.pdf]
