# Supplementary material for: Dissecting EXP2 sequence requirements for protein export in malaria parasites
Source: Front Cell Infect Microbiol. 2024 Jan 12;13:1332146. doi: 10.3389/fcimb.2023.1332146 (PMC10811066; doi:10.3389/fcimb.2023.1332146)

A

PfEXP2

MKVSYIFSFFLLFFVYKNTNTVVCDNGYGDLAATSALTTVIKDPISLTIKDIYEHGVKNPF

TKIIHKLKKFIRYRKVLRWSRMWWVLLVREIVGDNTIEKKTEKALREIWDQCTIAVYNN

TLNAVESKPLLFLHGILNECRNNFATKLRQDPSLIVAKIDQIIKSQIYRFWVSEPYLKIGRS

HTLYTHITPDAVPQLPKECTLKHLSSYMEEKLKSMESKKNIESGKYEFDVDSSETDSTKD

DGKPDDDDDDDDNFDDDDNFDDDTVEEEDASGDLFKNEKKDENKE

B

```
                                                 TMD Helix
GRA17-FL-aa-113-300 -YAEVAKVEALTNMISTPIELFVKDVWQQLFHKSGKPVWENMLFKFGSMIRHRAAHKATL
6E10_B              GYGDLAATSALTTVIKDPISLTIKDIYEH---GVK-NPFTKIIHKLKKFIRYRK------
                     *.::* ..***.:*. **.* :**::::         : :::.*: .:**:*

                        B1                          B2
GRA17-FL-aa-113-300 VIMWELRHFLYGTAKVNPSAWKKLETKFESYLREWWM------------TVPEDPWAALH
6E10_B              VLRWSRMWWVLLVREIVGD--NTIEKKTEKALREIWDQCTIAVYNNTLNAVESKPLLFLH
                    *: *.   ::  . ::  .  :.:*.* *. *** *              :* ..*   **

                         B3                         B4                   B5
GRA17-FL-aa-113-300 AGAWKSLLKLYNEDLEPLLRGSPKLK--DLESILFDSKLATIRRWTDEAHIEVMKG----
6E10_B              G-----ILNECRNNFATKLRQDPSLIVAKIDQI---IKSQIYRFWVSEPYLKIGRSHTLY
                    .     :*:  .:::   ** .*.*   .::.*    *    * *..* :::: :.

                                          LINKER HELIX
GRA17-FL-aa-113-300 --RTSNMVPR------LEALSAKMAVKQKAMQGKQ-------------------------
6E10_B              THITPDAVPQLPKECTLKHLSSYMEEKLKSMESKKNIESGKYEFDVDSSETDSTKDDGKP
                       * : **:      *: **: *  * *:*:.*:  * .* *  .:

GRA17-FL-aa-113-300 -----------------------------------------
6E10_B              DDDDDDDDNFDDDDNFDDDTVEEEDASGDLFKNEKKDENKE
```

C

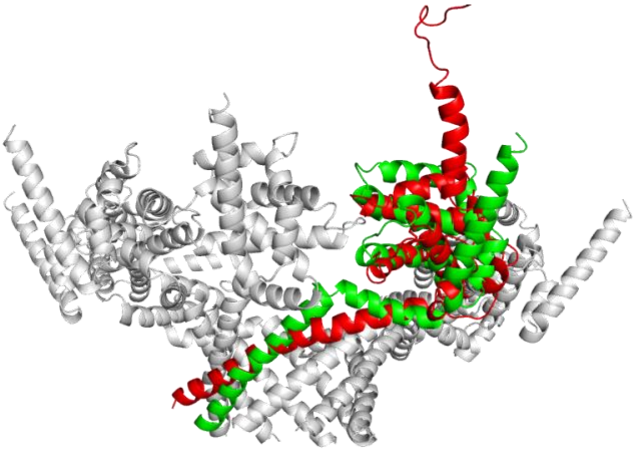

Supplement: Supplementary Figure 2 — Comparison between EXP2 and GRA17. (A) Coding sequence of EXP2, with the underlined sequence indicating the acidic tail (216-287). (B) Pairwise sequence alignment of GRA17 (56-243) and EXP2 (27-287) highlighting in bold the secondary structure elements. The underlined sequence represents the Acidic Tail of EXP2 (216-287) used in our experimental design. (B) AlphaFold model of GRA17 (56-243) in a heptamer assembly (grey)(Paredes-Santos et al., 2023). GRA17 monomer (green) aligned with a monomer of EXP2 (pdb 610, chain B, red). The model ends at EXP2 S235, so most of the AT is absent from this model. Alignment and schematic were generated in Pymol. [file DataSheet_2.pdf]
