# Supplementary material for: Dissecting EXP2 sequence requirements for protein export in malaria parasites
Source: Front Cell Infect Microbiol. 2024 Jan 12;13:1332146. doi: 10.3389/fcimb.2023.1332146 (PMC10811066; doi:10.3389/fcimb.2023.1332146)
Supplement: Supplementary Table 1 — Oligonucleotides used in this study. [file Table_1.docx]

**Table S1. Oligonucleotides used in this study.**

| **ID** | **Binding site** | **Name** | **Sequence (5’-3’)** |
| --- | --- | --- | --- |
| DO753 | PfEXP2 | PfEXP2_5’F NotISalI | TATGCGGCCGCGTCGACGCACCTAATTTAGTGGTTTCTT |
| DO757 | PfEXP2 | PfEXP2F-BglII | GGCAGATCTATGAAAGTCAGTTATATATTTTCCTTT |
| DO758 | PfEXP2 | PfEXP2R-Pst | GCGCTGCAGCTTCTTTATTTTCATCTTTTTTTTCATTTTTA |
| DO811 | cMYC tag | CMYC-INT_R | CTACAGGTCTTCCTCTGAAAT |
| DO862 | PfEXP2 | PfEXP2AT_F_AgeI | GCGACCGGTTCTATGGAATCAAAGAAAAATATAG |
| DO863 | PfEXP2 | PfEXP2AT_R_NheI | GCGGCTAGCTTCTTTATTTTCATCTTTTTTTTCATTTTTAA |
| DO891 | PfEXP2 | PfEXP2_AgeI_R | GCGACCGGTTTCTTTATTTTCATCTTTTTTTTCATTTTTAAATAA |
| DO1115 | PfEXP2 | C6_PfEXP2-AT_Rev | GCGACCGGTTTTCTTTGATTCCATAGATTTCAATTTC |
| DO1123 | PfHSP101 | C8_HSP101_F | CGCAGGCCTATGACAAGAAGATATTTAAAGTATTATATTTTTG |
| DO1124 | PfHSP101 | C8_HSP101_R | GCGACCGGTGGTCTTAGATAAGTTTATAACCAAG |
| DO1125 | PfClpB1 | C9_ClpB1_F | CGCGTCGACGCTTTATTTATGAGTGATGAAGAATATAC |
| DO1126 | PfClpB1 | C9+10_ClpB1_R | GCGCCTAGGACTTTTTGAAAAGTGCAACTTCTG |
| DO1127 | PfClpB1 | C10_ClpB1_F | CGCGGATCCCTAGAAAAATATAGTAGAGATCTAACAGCTTTG |
| DO1134 | PfHSP101 | PfHSP101_5'UTR_Fwd | TATGTATATATGACATCGTTAGTTCG |
| DO1167 | PfClpB1 | ClpB1_CDS_Fwd | GAGCAGCTGAATTGAGATTTG |
| DO1168 | PfClpB1 | ClpB1_CDS_Rev | CAAATCTCAATTCAGCTGCTC |
| DO1169 | PfHSP101 | 101_CDS_Fwd | GATTGGTGCAACAACTATAGC |
| DO1170 | PfHSP101 | 101_CDS_Rev | GCTATAGTTGTTGCACCAATC |
| DO1171 | PfHSP101 | 101_CDS_Fwd | GTGATGCTGTTGTGAAAGCAG |
| DO1172 | PfHSP101 | 101_CDS_Rev | CTGCTTTCACAACAGCATCAC |
| DO1173 | PfHSP101 | 101_CDS_Rev | CTACAGGTCTTCCTCTGAAAT |
| DO1191 | PfRESA | RESA_5'_Fwd | TATGTCGACATTTGTTCTTATTTATATACATTTCGTTTTAATTTAAG |
| DO1192 | PfRESA | RESA_3'_Rev | CGCAGATCTAATTATTTAGATATTTTCTTATTATAAATTATGTAAAAG |
| DO1219 | PfEXP2 | C11_swPCR_fwd | CACTGTAGTTGCTGATAACGGATATGGAGATTTAGC |
| DO1220 | PfEXP2 | C11_swPCR_rev | CCGTTATCAGCAACTACAGTGTTTGTATTTTTATATACG |
| DO1221 | PfEXP2 | C12_swPCR_fwd | CCAAAAGAAGCTACATTAAAACACTTATCATCCTATATG |
| DO1222 | PfEXP2 | C12_swPCR_rev | GTGTTTTAATGTAGCTTCTTTTGGTAATTGTGGTACAGC |
| DO1240 | PfHSP101 | PfHSP101_SeqOlg_F | GAAGTTTATTTAACCGATGAAGCC |
| DO1241 | PfHSP101 | PfHSP101_SeqOlg_R | GGCTTCATCGGTTAAATAAACTTC |
| DO1242 | PfClpB1 | PfClpB1_SeqOlg_R | CTTCTTTCTAATGCTTTATCCTTTTC |
| DO1243 | PfClpB1 | PfClpB1_SeqOlg_R | TGTTGGTCCTAAAAACATTAAAGATG |
| DO1247 | PfClpB1 | PfClpB1_SeqOlg_R | GCTTGATAAGTCATTTCTGGTG |
| DO1295 | TgGRA17 | TgGRA17_CDS_Fwd | GATCCGACACCGTGCAGCAC |
| DO1303 | PfEXP2 | PfEXP2_CDS_F | GGGATCAATGTACCATCGCTG |
| DO1314 | PfHSP101 | HSP101_Nterm_R | GCGGGATCCGTATAAAGTTCCGGTTTTTCCATC |
| DO1315 | PfHSP101 | HSP101-Nterm_F | TATGTCGACATTGAACAATTTGGTTCCAATATGAATG |
| DO1316 | PfHSP101 | HSP101_Seq_R | TCTTTGCTAGGGCACTTAAAATG |
| DO1317 | PfClpB1 | ClpB1-SS_Seq_R | CATCAATTTCTTGAACTAATAATTGGG |
| DO1318 | PfClpB1 | ClpB1-Nterm_Seq_R | CTTTTAATGAGTCAGGTACATCTC |
| DO1319 | PfHSP101 | HSP101_Seq_F | GAACCTACATAAAATCGTTGCATTAAG |
| DO1320 | PfRESA | RESA_Seq_F | CCTTATTCTTGGTTGTTATATATTTG |
| DO1321 | PfRESA | RESA_Seq_R | CAAATATATAACAACCAAGAATAAGG |
| DO1322 | PfRESA | RESA_Screen_F | AAAAATTACTTGGTTTTAATTTTTTACTTTTAC |
| DO1326 | PfEXP2 | EXP2_Scr_F | CGTTTCTGGGTATCAGAACCATAC |
| DO1327 | PfEXP2 | EXP2_Seq_F | CGTATGTGGTGGGTATTGTTAG |
| DO1328 | PfEXP2 | EXP2_Seq_R | CTAACAATACCCACCACATACG |
| DO1337 | PfHSP101 | PfHSP101_seq_R | GGGCACTTAAAATGTGTATAGG |
| DO1338 | PfClpB1 | PfClpB1_seq_R | AATAATTGGGTATCTATACCACTTTC |
| DO1339 | PfClpB1 | PfClpB1-N_seq_R | CAGGTACATCTCCTTGTACG |
| DO1340 | PfHSP101 | PfHSP101-N_Seq_R | CTTTTGGTACATCTCCTTTTTCAATTC |
| DO1376 | PfRESA | RESA5’_8_F | ATAGTCGACTCTATTTGTTCTTATTTATATACATTTCG |
| DO1377 | PfRESA | RESA5’_8_R | GCGAGGCCTAATTATTTAGATATTTTCTTATTATAAATTATGTAAAAG |
| DO1378 | PfRESA | RESA5’_9+14_F | ATACCCGGGTCTATTTGTTCTTATTTATATACATTTCG |
| DO1379 | PfHSP101 | HSP101ss_9+14_R | CGCGTCGACATCGGGAGCACACAATACATTAT |
| DO1380 | PfEXP2 | EXP2cds_Scr_R | CGTTATCACAAACTACAGTGTTTG |
| DO1381 | PfHSP86 | HSP86-5’_Scr_F | GTATTTACAAAATATAATAAAATAATTTCATGTTTAGCAAAAAT |
| DO1382 | TgGRA17 | GRA17cds_Scr_R | CGAATTGCTCGCATAGATCT |
| DO1810 | PfFBPA | FBPA_F | TGTACCACCAGCCTTACCAG |
| DO1811 | PfFBPA | FBPA_R | TTCCTTGCCATGTGTTCAAT |
| DO1812 | PfEXP2 | EXP2-RT_F1 | GTGGTGGGTATTGTTAGTAAGAG |
| DO1813 | PfEXP2 | EXP2-RT-F2 | GTGGCAAAGTTGTTTCTGCATTC |
| DO1869 | PfEXP2 | EXP2-RT_F2 | GATACCGTTGAAGAAGAAGATGC |
| DO1870 | cMYC tag | cMYC-RT_R | CTACAGGTCTTCCTCTGAAATGAG |
