# Supplementary material for: Dissecting EXP2 sequence requirements for protein export in malaria parasites
Source: Front Cell Infect Microbiol. 2024 Jan 12;13:1332146. doi: 10.3389/fcimb.2023.1332146 (PMC10811066; doi:10.3389/fcimb.2023.1332146)
Supplement: Supplementary Table 2 — The fold changes of exp2-cMyc expression under the ptex150 promoter relative to the resa promoter. [file Table_2.docx]

**Table S2: The fold changes of *exp2-cMyc* expression under the *ptex150* promoter relative to the *resa* promoter**

| **Parasite stage** | **Transcript** | | **Protein** | |
| --- | --- | --- | --- | --- |
|  | Total *exp2* | *exp2-cMyc* | EXP2-HA | EXP2-cMyc |
| Rings | 1.22 | 2.47 | 1.00 | 2.04 |
| Trophozoites | 0.98 | 4.68 | 0.52 | 1.54 |
| Schizonts | 1.03 | 1.5 | 1.09 | 1.82 |
